# Diagnosis of Syphilis in Paraffin-Embedded Skin Biopsies: Comparison of *Treponema pallidum* Immunohistochemistry and Polymerase Chain Reaction in Primary and Secondary Stages of Disease

**DOI:** 10.3390/dermatopathology13030029

**Published:** 2026-07-02

**Authors:** Charlotte C. Fuchs, Bastian Stoffers, Stephan A. Braun, Almut Böer-Auer

**Affiliations:** 1 Department of Dermatology, University Hospital Münster, Von-Esmarch-Straße 58, 48149 Münster, Germany; stephanalexander.braun@ukmuenster.de (S.A.B.); boer@dermatologikum.de (A.B.-A.); 2 Dermatologikum Hamburg, Stephansplatz 5, 20354 Hamburg, Germany; bastian.stoffers@dermatologikum.de; 3 Department of Dermatology, Medical Faculty, Heinrich-Heine University, Moorenstraße 5, 40225 Düsseldorf, Germany

**Keywords:** syphilis, immunohistochemistry, PCR, *Treponema pallidum*, plasma cells, CD 138

## Abstract

Syphilis remains one of the most prevalent sexually transmitted infections. Serology is the diagnostic gold standard, but skin biopsies aid in ambiguous cases. Treponemas can be detected in tissue by immunohistochemistry (IHC) and polymerase chain reaction (PCR); however, their comparative performance across disease stages has not been extensively studied. This retrospective study of 48 formalin-fixed, paraffin-embedded (FFPE) skin biopsies from syphilis cases compared *Treponema pallidum* IHC and PCR. Of 48 biopsies, 42 (88%) were positive by *T. pallidum*–specific PCR, whereas IHC identified 45 (94%; *p* = 0.04). Organisms were denser in primary syphilis (*p* = 0.03) and predominantly involved the lower third of the epidermis. In 7 of 45 biopsies (15%), treponemas were only detected in the dermis (all secondary syphilis). Therefore, *Treponema pallidum* IHC demonstrated a slight advantage over PCR, but low Treponema density in about 40% and epidermal absence in nearly 20% of secondary syphilis biopsies highlight a substantial risk of overlooking treponemas on IHC. Systematic assessment including endothelium and perivascular areas is essential.

## Simple Summary

Syphilis remains a prevalent sexually transmitted infection. It can mimic inflammatory or neoplastic disorders, occasionally prompting biopsy without clinical suspicion. In formalin-fixed skin biopsies, immunohistochemistry and PCR can confirm *Treponema pallidum* in the tissue. We compare results of both methods in 48 biopsies of patients with syphilis at different stages of disease. In our patients, immunohistochemistry performed slightly better than PCR; however, identification of organisms on immunohistochemistry remained challenging in some cases with sparse organisms. We analyzed the distribution of *Treponema pallidum* in the tissue in order to facilitate diagnosis, particularly in secondary syphilis when low organism burden and absence of organisms from the epidermis may lead to misdiagnosis. In such cases, PCR is helpful to confirm the diagnosis.

## 1. Introduction

Syphilis, caused by *Treponema pallidum*, is a common sexually transmitted infection with rising global incidence [1]. In Germany, new cases have surged post-coronavirus pandemic, particularly among high-risk groups [2]. Spirochetes are transmitted sexually, via blood, or congenitally [3]. Untreated, it progresses to chronic disease with a risk of involvement of multiple organs, such as the cardiovascular system, spinal cord, or eyes.

Disease stages include primary, secondary, latent, and tertiary syphilis. The primary chancre (ulcus durum) appears days to weeks post-infection, typically genitoanally. Secondary syphilis arises via hematogenous dissemination, often with systemic symptoms (fever, lymphadenopathy, fatigue) and a trunk-centered maculopapular exanthem involving palms and soles [3]. Persistent infection may lead to tertiary syphilis with gummatous lesions in any organ [3].

Although serology is highly sensitive and represents the cornerstone of syphilis diagnosis, its interpretation may be challenging in previously infected patients [4]. Persistently positive treponemal tests and residual non-treponemal titers may obscure the distinction between past treated infection, active disease, and reinfection. Therefore, serology has important limitations in this specific setting.

In clinically equivocal skin lesions, biopsies are often performed to confirm or exclude differentials. Since syphilis can mimic other inflammatory and neoplastic conditions, in some cases the disease is not considered clinically, but the suspicion of the infection is rendered due to suggestive patterns and composition of infiltrates in the biopsy. Immunohistochemical (IHC) and molecular methods by polymerase chain reaction (PCR) are available to confirm syphilis in Formalin-fixed, Paraffin-embedded (FFPE) biopsy material. So far, the literature provides only very limited information on the diagnostic value and comparative performance of IHC and PCR in FFPE specimens of syphilis along the different stages of disease [5,6,7,8]. This study compares the diagnostic performance of *Treponema pallidum*–specific immunohistochemistry and *Treponema pallidum*–specific PCR in the diagnosis of syphilis in FFPE skin biopsies. Two PCR assays with different target genes were utilized in a two-step approach, a conventional PCR, which amplifies either a 379-bp or a 196-bp fragment, and a real-time PCR (qPCR), targeting a 72-bp fragment.

Moreover, we attempted to determine histopathological patterns and clinicopathological constellations in which either *Treponema pallidum*–specific immunohistochemistry or *Treponema pallidum*–specific PCR is more likely to be successful in confirming the infection.

## 2. Material and Methods

### 2.1. Study Population

This study analyzed 48 FFPE skin biopsies from 38 consecutive adults diagnosed with syphilis at our institutes in a retrospective setting. Inclusion in the cohort required at least one positive *Treponema pallidum* PCR or an unequivocally positive result in the *Treponema pallidum* IHC (one biopsy in 28 patients; two biopsies in 10 patients). 44 biopsies stem from male and 4 from female patients. The age range was between 22 and 74 years. Clinical data, e.g., diagnoses and differential diagnoses, serological confirmation, were derived from patient files and photographs. Twelve biopsies from primary syphilis and 36 biopsies from secondary syphilis were included. No patient had tertiary syphilis. In case of clinicopathological correlation, all patients were assigned to the stage of disease according to current criteria of the European guideline [9].

All biopsies were examined histologically, with *Treponema pallidum* IHC and with assays targeting *Treponema* pallidum–specific DNA. In a subset of cases with sufficient material, immunophenotyping of the infiltrate was performed. Two investigators (A.B., C.F.) independently assessed specimens, resolving ambiguities by consensus.

All patients had given informed consent to the biopsy procedure at the time of diagnosis. All data were anonymized prior to analysis. The study was conducted in accordance with the principles outlined in the Declaration of Helsinki (1975, revised in 2013) and approved by the ethics committees of the States of Hamburg (2024-300484-WF), approval date: 30 May 2024) and Nordrhein-Westfalen (2024-573-f-S), approval date: 7 October 2024), Germany.

### 2.2. DNA Isolation

Total DNA was extracted from FFPE skin biopsies using 10 to 20 tissue sections (5 µm thickness). Nucleic acid extraction was performed with the MagNA Pure 96 DNA and Viral NA Small Volume Kit (Roche, Mannheim, Germany). Paraffin blocks lacking tissue served as negative and cross-contamination controls. Amplification of the ß-actin gene was employed as internal quality control.

### 2.3. Treponema pallidum–Specific Polymerase Chain Reaction (PCR)

Two PCR assays were utilized for the detection of *Treponema pallidum*: a conventional PCR, which amplifies either a 379-bp or a 196-bp fragment, and is analyzed using capillary electrophoresis following amplification, thereby yielding a qualitative or semi-quantitative result. Conversely, the second test was a real-time PCR (qPCR), which employs fluorescently labeled probes to target a 72-bp fragment, thereby enabling quantitative detection in real time by measuring the rise in the fluorescent signal during each cycle.

The amplification of the 379 bp target region was carried out using the primer pair 5′-CAGCAGGGGAAGAAAAAAGTGGG-3′ (forward) and 5′-AAGGTCGTGCGGGCTCTCCAT-3′ (reverse).

The following primers were used for the amplification of the 196 bp segment: 5′-GACCCAAGCGTTACTAAGATGG-3′ (forward) and 5′-ACCGCAACTGGGACAAACTTCAT-3′ (reverse) (PCR-2 Primer [10]).

The amplification of the β-actin gene was utilized as an internal quality control measure. The following primers were employed for this purpose 5-TCACCCACACTGTGCCCATCTACGA-3’ (forward); 5-CAGCGGAACCGCTCATTGCCAATGG-3′ (reverse) [11].

PCR reactions were conducted in 12 µL volumes containing 7 µL HotStarTaq Master Mix (Qiagen, Hilden, Germany) and 5 µL of total extracted DNA. The Master Mix consists of 180 µL MM + 8 µL forward and 8 µL reverse primers.

The thermal cycling program started with an initial denaturation step at 95 °C for 15 min, followed by 40 cycles of 94 °C for 20 s, 65 °C for 30 s, and 72 °C for 30 s. This was followed by a single elongation step at 72 °C for 5 min. Subsequently, 25 additional cycles were performed comprising 94 °C for 20 s, 63 °C for 1 min, and 72 °C for 30 s, with a final elongation at 72 °C for 15 min.

The analytical separation of the PCR products is then carried out by capillary electrophoresis with the QIAxcel Advanced System (Qiagen, Hilden, Germany).

In 11 cases with positive IHC in which the conventional assay was negative, the second approach, a real-time PCR assay (qPCR), according to Chen and Pilay [12], was used if enough material was left.

The following primers and a fluorescent-labeled probe were used for the amplification of the 72 bp target region: Forward: 5′CAGGATCCGGCATATGTCC3′; Reverse: 5′AAGTGTGAGCGTCTCATCATTCC3′; Probe: 5′FAM-CTGTCATGCACCAGCTTCGACGTCTT-BHQ1′.

The thermal cycling program started with an initial denaturation step at 95 °C for 10 min, followed by 45 cycles of 95 °C for 20 s, annealing at 60 °C for one minute, and extension at 60 °C for one minute.

The amplification of the human ribonuclease P gene (RNase P) was utilized as an internal quality control measure. The following primers were employed for this purpose:

Forward: 5′-CCAAGTGTGAGGGCTGAAAAG-3′

Reverse: 5′-TGTTGTGGCTGATGAACTATAAA AGG-3′

Probe: 5′-CY5-CCCCAGTCTCTGTCAGCACTCCCTTC-BHQ-650-3′

### 2.4. Treponema pallidum–Specific Immunohistochemistry

Sections were stained with *Treponema pallidum*–specific antibodies (polyclonal), rabbit IgG1 (DCS Innovative Diagnostics Systems), dilution 1:50 with EnVision Flex Antibody Diluent (Agilent), including positive and negative controls. Sections were interpreted as positive when stained structures exhibited spirochetal morphology. Sections were examined for the localization of Treponema in the epidermis (lower, mid, upper part of it), in adnexal epithelia, in endothelia of superficial and deep plexus, in perivascular connective tissue and in the interstitial dermis. A semi-quantitative score (0: none, 1: few, <10 per High-power-field (HPF), 2: moderate, 10–50, 3: numerous, >50 per HPF) was used to evaluate the density of organisms, as previously used by Müller et al. [5].

### 2.5. Histopathology and Immunophenotyping

Hematoxylin and Eosin (HE)-stained sections were examined for epidermal changes as well as patterns and composition of the dermal infiltrate (absence vs. presence or semi-quantitative score: 0–3: none, little/few, moderate, dense/numerous). In 26 biopsies, material was sufficient to phenotype the infiltrate by immunohistochemistry with the following markers: CD3 (FLEX Polyclonal Rabbit Anti-Human CD3 clone: F7.2.38), CD20 (Monoclonal Mouse Anti-Human CD20cy, Clone L26), CD138 (FLEX Monoclonal Mouse Anti-Human CD138, Clone MI15), CD68 (FLEX Monoclonal Mouse Anti-Human CD68, Clone PG-M1), CD34 (FLEX Monoclonal Mouse Anti-Human CD34 Class II, Clone QBEnd 10), all from Agilent (Agilent Technologies Denmark ApS, Glostrup, Denmark), and CD123 (Invitrogen CD123 Monoclonal Antibody, Clone 6H6) from eBioscience, Life Technologies, Thermo Fisher Scientific, Darmstadt, Germany. All markers were assessed for absence vs. presence or with a semi-quantitative score: 0–3.

### 2.6. Statistics

Selected parameters were assessed for statistical significance using Fisher’s exact test (2-tailed) or Mann–Whitney U test (2-tailed) for semi-quantitative data. Statistical significance was assessed at the 95% significance level. Descriptive statistics were used to compare multiple morphological features.

Primary endpoints: positive PCR and positive IHC in biopsies of syphilis according to stage.

Secondary endpoints: Differences in distribution of organisms, histomorphologic and immunohistochemical features according to stage.

## 3. Results

Demographical data, clinical diagnosis and differential diagnosis, and serological confirmation are available in Table 1 together with molecular and immunohistochemical results of all biopsies.

### 3.1. Treponema pallidum PCR Assays

42 out of 48 skin biopsies (88%) were tested positive for *Treponema pallidum*–specific DNA in at least one of the PCR assays. The conventional PCR assay yielded a positive result in 35 biopsies (35 of 48; 73%), while the real-time PCR assay identified *Treponema pallidum* in 7 more biopsies. 4 biopsies were found to be negative in both assays.

For 2 additional biopsies with negative PCR results, only the conventional PCR approach was applied due to insufficient material. All of these cases were assigned to secondary syphilis.

### 3.2. Treponema pallidum Immunohistochemistry

Applying immunohistochemistry (IHC), *Treponema pallidum* was detected in the epidermis and/or dermis in 45 of 48 biopsies (94%). *Treponema pallidum* was detected in 100% of cases of primary syphilis and 92% in cases of secondary syphilis.

Interestingly, treponemas were identified in the epidermis in just 40 of 48 biopsies (83%) (including one case that was entirely ulcerated so that epidermis could not be assessed), and again less often in secondary syphilis (81% vs. 92%).

**Figure 1:**
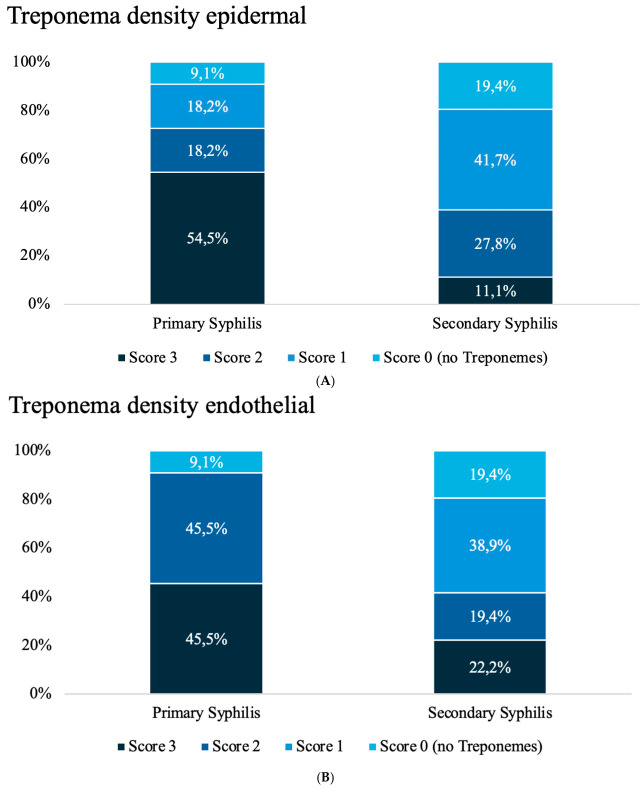
(**A**): Epidermal density of Treponema in skin biopsies of primary and secondary syphilis. (**B**): Treponema density in endothelium of blood vessels in skin biopsies of primary and secondary syphilis.

**Figure 2:**
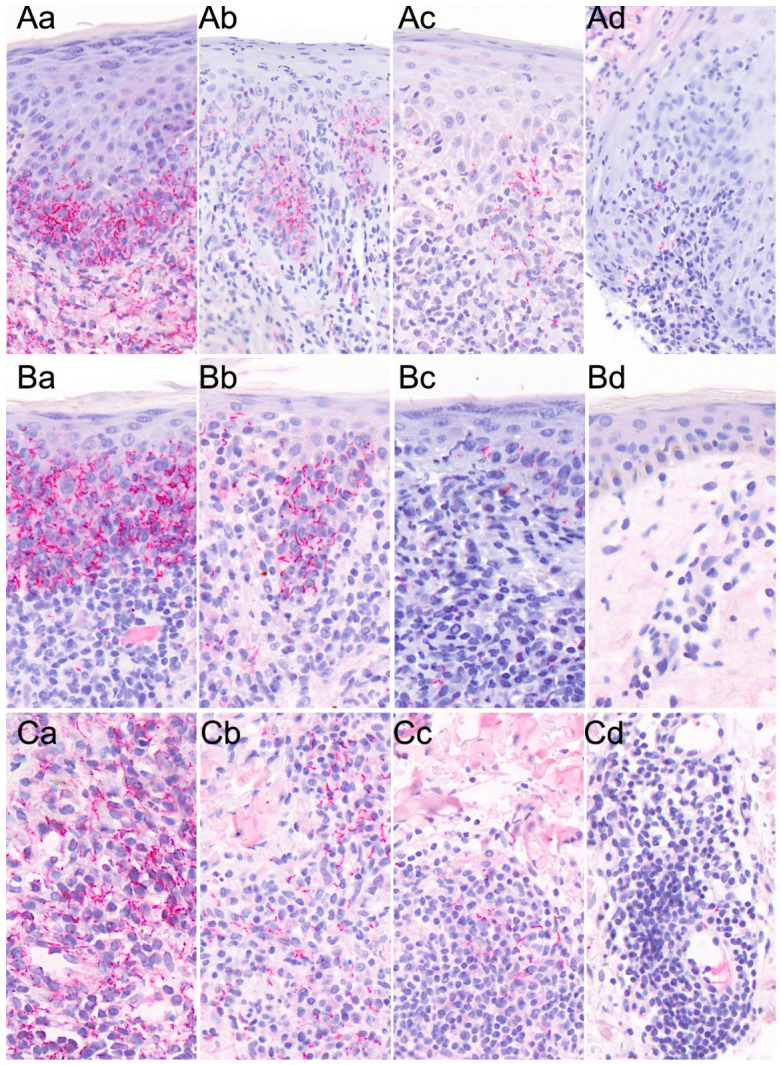
Treponema pallidum immunohistochemistry. Density and distribution of organisms. (**Aa**–**Ad**) Epidermal Treponemas in primary syphilis. Lower third of the epidermis consistently emphasized. (**Aa**) Dense (Score 3); (**Ab**) Moderate (Score 2); (**Ac**) Sparse (Score 1); (**Ad**) Sparse with very few Treponemas in a case of reinfection. (**Ba**–**Bd**) Epidermal Treponemas in secondary syphilis. (**Ba**) Dense lower and mid third of the epidermis; (**Bb**) Moderate; (**Bc**) Sparse; (**Bd**) Absence of Treponemas in the epidermis in a case of reinfection. (**Ca**–**Cd**) Dermal Treponemas. (**Ca**) Primary syphilis, dense perivascular; (**Cb**) Secondary syphilis, moderate perivascular; (**Cc**) Secondary syphilis, few perivascular; (**Cd**) Secondary syphilis in a case of reinfection, very few organisms in the endothelium. In the dermis, treponemas were predominantly localized in endothelial cells and around vessels (39 of 47, 82%, and 38 of 47, 80%). In contrast, their presence in the interstitial dermis was less frequent (26 of 47; 55%). A high density (score 3) was identified in 33% of primary syphilis biopsies but not in any of the secondary syphilis biopsies (*p* < 0.05).

In the epidermis, treponemas were located mostly in the lower third (40 of 47; 85%) while the mid and upper epidermis were involved much less commonly (51% and 19%). The Treponema location differed between primary and secondary syphilis. The mid and upper epidermis were involved in 64% and 55% of primary syphilis biopsies but in only 47% and 8% of secondary syphilis biopsies (*p* < 0.05). Moreover, there were differences in density of organisms, e.g., high density (score 3) was identified in the lower third of the epidermis in 45% of primary syphilis biopsies but in only 11% of secondary syphilis biopsies (Figure 1). Notably, 19% of secondary syphilis biopsies did not show any treponemas in the epidermis (Table 2, Figure 2). Immunohistochemical assessment and results are illustrated in Figure 2.

Comparison of primary and secondary syphilis biopsies revealed that perivascular and endothelial involvement was 10% higher in primary compared to secondary syphilis biopsies. Furthermore, density of organisms varied significantly, with a score of 3 found around vessels and in endothelial cells in 50% of primary syphilis biopsies, but only 11% and 28% of secondary syphilis (*p* < 0.05) (Table 2).

In three out of 48 biopsies, *Treponema pallidum* could not be visualized using IHC. All of these cases were secondary syphilis, two coming from the same patient. In 17 additional biopsies, only very sparse epidermal and dermal spirochetes were detected: 15 of them were secondary syphilis, including one case of reinfection in secondary syphilis; the remaining 2 came from a patient with primary syphilis as a reinfection. In four patients, both biopsies taken simultaneously demonstrated only very sparse organisms.

A total of 8 biopsies revealed dense epidermal treponemas. Five of these came from patients with primary syphilis, of which three also contained dense organisms in the dermis, while two showed a moderate dermal treponemal density (score 2). Of note, three biopsies from secondary syphilis, including one case of reinfection, exhibited plenty of epidermal treponemas, while all other biopsies in this group revealed only sparse organisms. However, the second biopsy taken simultaneously from the case of reinfection showed only sparse treponemas in both the epidermis and dermis, meaning that even in one and the same patient at the same point in time the density of organisms in lesional tissue may be variable.

### 3.3. Comparison of Immunohistochemistry and Molecular Testing of Treponema pallidum

In this study, IHC was slightly more effective than PCR in detecting treponemas and diagnosing syphilis infection in FFPE skin biopsies (*p* = 0.04), even though we utilized the two-step PCR testing protocol, which yielded a positive PCR result in seven additional biopsies after the first assay had been negative. However, interpretation of IHC also was challenging due to the variable density and distribution of the organisms in the tissue.

Comparing the results of IHC and PCR, we found that a total of 6 of 48 (12,5%) biopsies, all classified as secondary syphilis, had negative results in the PCR assay. Notably, two of the biopsies came from a patient who showed very sparse treponemas on IHC. In the remaining cases, an additional biopsy had been obtained concurrently, yielding a positive PCR. Four of the PCR-negative biopsies exhibited a positive result in IHC, but pathogen density remained low in all these cases.

In two additional biopsies, both PCR and IHC returned negative, while the patients had a PCR- and/or IHC-positive second biopsy from the same skin eruption at the same time.

### 3.4. Histopathology and Immunophenotype

An overview of histopathological findings is provided in Table 3A–C. As expected, ulceration and erosion were predominantly observed in biopsies of primary syphilis (*p* < 0.05), but interestingly, epidermal atrophy was observed in 14 of 48 (29%) biopsies, 12 of which were from secondary syphilis cases. Necrotic keratinocytes were present in 60% of biopsies, and intraepidermal inflammatory cells, as well as a subepidermal band-like infiltrate, were detected in the vast majority of cases. In secondary syphilis, a perivascular pattern dominated (97%), whereas in primary syphilis, a diffuse pattern was significantly more frequent (*p* < 0.05).

Infiltrates in primary syphilis were almost always dense (score 3), a feature observed in only one case of secondary syphilis.

Regarding the composition of the inflammatory infiltrate, lymphocytes and plasma cells were identified in 98% of biopsies irrespective of disease stage.

In contrast, neutrophilic granulocytes had a stage-dependent variation (primary syphilis: 11 of 12; 92% vs. secondary syphilis: 20 of 36; 56% of biopsies (*p* < 0.05). Macrophages were consistently observed, displaying a diffuse pattern in 15 of 25 60% of cases; 70% primary syphilis and 53% secondary syphilis) and with well-formed granulomas in 23% of biopsies (primary syphilis: 17%; secondary syphilis: 25%).

Immunohistochemical staining (*n* = 26) revealed a T-cell-dominant infiltrate throughout the cohort.

B-cells were present in 21 of 26 cases (81%) but were detected in only 5 of 10 biopsies of primary syphilis (50%), compared with all biopsies from secondary syphilis (16 of 16; 100%; *p* < 0.05).

**Figure 3:**
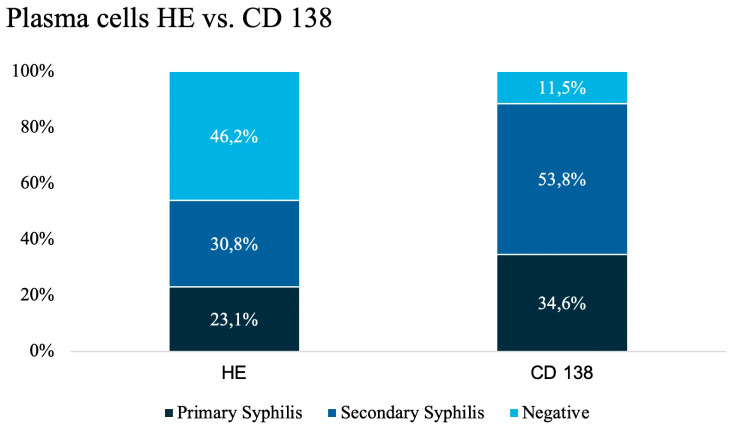
Comparison of detection of plasma cells in hematoxylin and eosin (HE) staining versus immunohistochemistry with CD138 antibodies.

In both stages, plasma cells were identified by CD138 staining in almost 90% of biopsies. However, plasma cells were absent in 10% and notably sparse in more than one-third of cases, particularly in secondary syphilis (3 of 10 in primary; 7 of 16 in secondary syphilis). A score 3 plasma cell infiltrate was observed in 50% of primary syphilis biopsies but in only 6% of secondary syphilis cases (*p* < 0.05). Comparison of hematoxylin–eosin (HE) assessment with CD138 staining demonstrated that identification of plasma cells increased from 54% (HE) to 88% of biopsies when using CD138 (Figure 3).

### 3.5. Clinical Correlation

**Figure 4:**
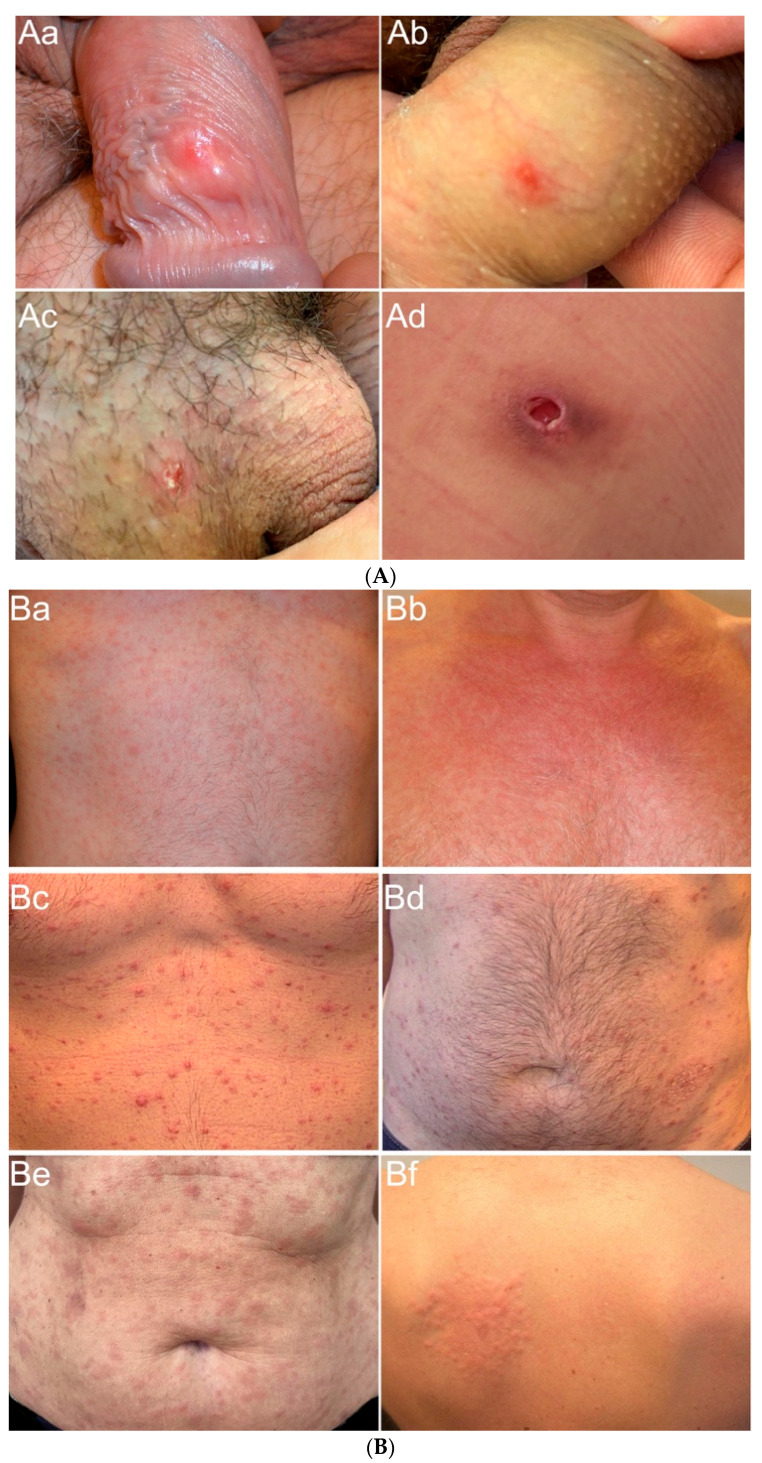
(**Aa**–**Ad**): Clinical diversity of primary syphilis. (**Aa**) Small asymptomatic nodule with tiny erosion on the penis shaft; (**Ab**) Erythematous macule, slightly erosive, on the penis shaft; (**Ac**) Polycyclic small and superficial ulceration on the mons pubis; (**Ad**) Ulcerated brownish nodule on the side of the trunk. (**Ba**–**Bf**) Clinical diversity of secondary syphilis. (**Ba**) Urticarial exanthem; (**Bb**) Patchy and confluent erythema on the chest; (**Bc**) Polymorphic exanthem simulating Pityriasis lichenoides acuta; (**Bd**) Exanthem of scaly papules and plaques reminiscent of psoriasis or pityriasis rosea; (**Be**) Brownish patchy exanthem; (**Bf**) Plaque of confluent papules in a HIV-positive patient.

Clinical features of our patients are illustrated in Figure 4A,B, showing the clinical diversity of lesions in primary and secondary syphilis.

**Figure 5:**
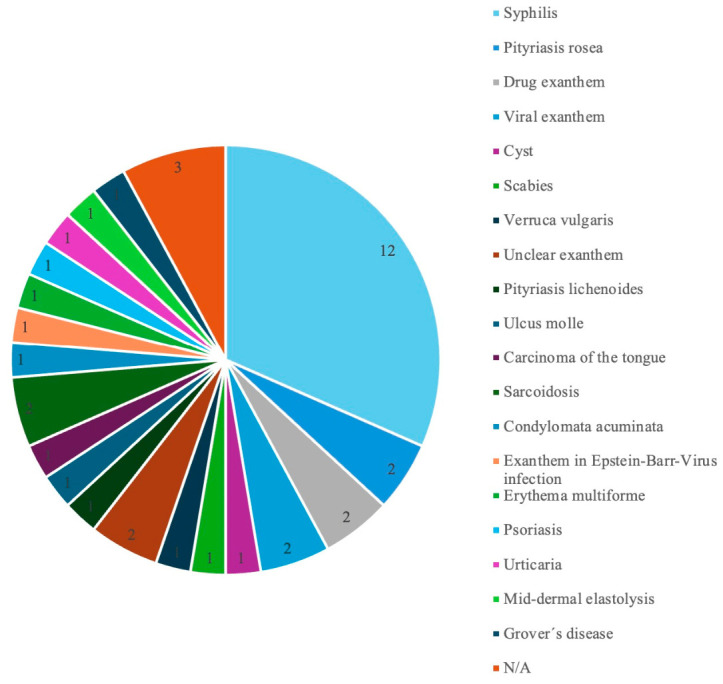
Clinical diagnosis in 38 patients with confirmed syphilis.

Syphilis was listed as the primary clinical diagnosis in only 12 of 38 patients (32%). Among these, four patients had primary syphilis, and seven had secondary syphilis. Frequently suggested diagnoses included pityriasis rosea (2 of 38; 5%), drug exanthem (2 of 38; 5%), and viral exanthem (2 of 38; 5%) (Figure 5). In 9 out of 38 patients (24%), syphilis was considered at least as a differential diagnosis. Among these, two patients had primary syphilis, and seven had secondary syphilis. Other recurrent differential diagnoses included pityriasis rosea (2 of 36; 6%) and psoriasis (3 of 36; 8%). For 12 out of 38 patients (32%), no diagnosis or differential diagnosis was provided. Overall, in nearly half of the study population (18 of 38; 47%), syphilis was not considered by the clinicians.

**Table 3:** (**A**–**C**). Histopathology (HE) in Biopsies of primary and secondary syphilis. (**A**) Epidermal Patterns; (**B**) Dermal Patterns; (**C**) Composition of Infiltrates. Score 1 (mild/few), 2 (moderate), 3 (marked/numerous).

**A: Epidermal Patterns**				
	**Syphilis Stages**			**Statistics**
	**1 + 2**	**1**	**2**	
**Epidermis**	**48/48 (100,0%)**	**12/12 (100,0%)**	**36/36 (100,0%)**	**n/s**
**Ulceration**	**7/48 (14,6%)**	**6/12 (50,0%)**	**1/36 (02,8%)**	**0,0005**
Score 1	0 (00,0%)	0 (00,0%)	0 (00,0%)	
Score 2	4 (08,3%)	3 (25,0%)	1 (02,8%)	
Score 3	3 (06,3%)	3 (25,0%)	0 (00,0%)	0,01278
**Erosion**	**9/48 (18,8%)**	**6/12 (50,0%)**	**3/36 (08,3%)**	**0,00420**
Score 1	5 (10,4%)	3 (25,0%)	2 (05,6%)	
Score 2	4 (08,3%)	3 (25,0%)	1 (02,8%)	
Score 3	0 (00,0%)	0 (00,0%)	0 (00,0%)	0,03
**Acanthosis**	**18/48 (37,5%)**	**7/12 (58,3%)**	**11/36 (30,6%)**	
Score 1	11 (22,9%)	4 (33,3%)	7 (19,4%)	
Score 2	4 (08,3%)	1 (08,3%)	3 (08,3%)	
Score 3	3 (06,3%)	2 (16,7%)	1 (02,8%)	
**Spongiosis**	**24/48 (50,0%)**	**7/12 (58,3%)**	**17/36 (47,2%)**	
Score 1	15 (31,3%)	4 (33,3%)	11 (30,6%)	
Score 2	9 (18,8%)	3 (25,0%)	6 (16,7%)	
Score 3	0 (00,0%)	0 (00,0%)	0 (00,0%)	
**Necrotic** **keratinocytes**	**29/48 (60,4%)**	**9/12 (75,0%)**	**20/36 (55,6%)**	
Score 1	24 (50,0%)	6 (50,0%)	18 (50,0%)	
Score 2	4 (08,3%)	2 (16,7%)	2 (05,6%)	
Score 3	1 (02,1%)	1 (08,3%)	0 (00,0%)	
**B: Dermal Patterns**				
**Perivascular**	**42/48 (87,5%)**	**7/12 (58,3%)**	**35/36 (97,2%)**	**0,00024**
Score 1	9 (18,8%)	1 (08,3%)	8 (22,2%)	
Score 2	22 (45,8%)	2 (16,7%)	20 (55,6%)	
Score 3	11 (22,9%)	4 (33,3%)	7 (19,4%)	
**Interstitial**	**36/48 (75,0%)**	**8/12 (66,7%)**	**28/36 (77,8%)**	
Score 1	24 (50,0%)	5 (41,7%)	19 (52,8%)	
Score 2	11 (22,9%)	2 (16,7%)	9 (25,0%)	
Score 3	1 (02,1%)	1 (08,3%)	0 (00,0%)	
**Nodular**	**12/48 (25,0%)**	**4/12 (33,3%)**	**8/36 (22,2%)**	
Score 1	2 (04,2%)	0 (00,0%)	2 (05,6%)	
Score 2	6 (12,5%)	1 (08,3%)	5 (13,9%)	
Score 3	4 (08,3%)	3 (25,0%)	1 (02,8%)	
**Diffuse**	**6/48 (12,5%)**	**5/12 (41,7%)**	**1/36 (02,8%)**	**0,00240**
Score 1	0 (00,0%)	0 (00,0%)	0 (00,0%)	
Score 2	1 (02,1%)	1 (08,3%)	0 (00,0%)	
Score 3	5 (10,4%)	4 (33,3%)	1 (02,8%)	
**C: Composition of Infiltrates**				
**Lymphocytes**	**47/48 (97,9%)**	**11/12 (91,7%)**	**36/36 (100,0%)**	
Score 1	9 (18,8%)	1 (08,3%)	8 (22,2%)	
Score 2	32 (66,7%)	9 (75,0%)	23 (63,9%)	
Score 3	6 (12,5%)	1 (08,3%)	5 (13,9%)	
**Plasma cells**	**47/48 (97,9%)**	**12/12 (100,0%)**	**35/36 (97,2%)**	
Score 1	22 (45,8%)	4 (33,3%)	18 (50,0%)	
Score 2	10 (20,8%)	2 (16,7%)	8 (22,2%)	
Score 3	15 (31,3%)	6 (50,0%)	9 (25,0%)	
**Neutrophilic** **granulocytes**	**31/48 (64,6%)**	**11/12 (91,7%)**	**20/36 (55,6%)**	**0,03540**
Score 1	20 (41,7%)	4 (33,3%)	16 (44,4%)	
Score 2	8 (16,7%)	4 (33,3%)	4 (11,1%)	
Score 3	3 (06,3%)	3 (25,0%)	0 (00,0%)	0,00174
**Histiocytes**	**45/48 (93,8%)**	**11/12 (91,7%)**	**34/36 (94,4%)**	
Score 1	15 (31,3%)	2 (16,7%)	13 (36,1%)	
Score 2	22 (45,8%)	7 (58,3%)	15 (41,7%)	
Score 3	8 (16,7%)	2 (16,7%)	6 (16,7%)	

## 4. Discussion

This study analyzes the diagnostic performance of *Treponema pallidum*–specific IHC and *Treponema pallidum*–specific PCR on FFPE biopsies in primary and secondary syphilis. Furthermore, stage-specific differences in pathogen localization and density as well as infiltrate character and immunophenotype were investigated. In our cohort of FFPE skin biopsies, IHC demonstrated slightly superior diagnostic performance compared with PCR (94% vs. 88%) in the detection of syphilis. Nevertheless, identification via IHC was challenging because of the heterogeneous density and distribution of organisms within the tissue. PCR, as well as clinical correlation and serology, were of help in confirming the diagnosis in such cases, so all available diagnostic methods should complement each other in challenging cases.

Reported sensitivities for PCR amount to 42% to 75% [5,6,7] and for IHC to 49% to 91% [5,7,8].

A study by Müller et al. [5] investigated PCR and IHC in skin biopsies in comparison with so-called Focus-floating microscopy, which was suggested at that time as an effective diagnostic method, but since then has not found its way into daily routine diagnostics in most dermatopathology laboratories. The results of conventional IHC and PCR in this study reached only 49% and 69%, respectively, giving the impression that these methods do not work well on FFPE. In our cohort, both methods performed much better, at 94% and 88%, respectively, showing that they both are important diagnostic tools in dermatopathology routine. However, Müller et al. [5] demonstrated that the sensitivity of both techniques decreased with advancing stage, an observation confirmed in our study. Similar to our findings, Müller et al. [5] found PCR consistently positive in primary syphilis biopsies, whereas negative PCR results occurred only in secondary syphilis. The investigations conducted by Buffet et al. [7] and Wenhaia et al. [6] exclusively included patients with secondary syphilis, which likely accounts for the lower PCR effectiveness in their study.

In contrast to other studies, our PCR employed two assays targeting different genes. This two-step PCR approach increased the number of positive specimens from 35 to 42; however, 6 of 48 biopsies (12%) remained PCR-negative. These biopsies also had a low pathogen density on IHC, which provides some explanation for the PCR negativity but shows that a negative PCR result does not exclude a syphilis infection. Nevertheless, combining PCR assays targeting different genes improved the overall diagnostic yield in syphilis [13]^,^ consistent with previous findings in PCR diagnostics of other spirochaetal infections using FFPE tissue [14]. It has been suggested that PCR may remain positive after effective treatment due to detection of residual DNA from non-viable spirochaetes; however, Wicher et al. [15] demonstrated in an animal model that dead spirochaetes were cleared from the organism within 15–30 days, indicating that a positive PCR result can be regarded as evidence of an active infection.

The existing literature provides limited data on differences in *Treponema pallidum* IHC across the various stages of syphilis [5,7,8], e.g., studies by Buffet [7] and Hoang [8], which concentrated exclusively on secondary syphilis. Therefore, we want to emphasize the marked stage-dependent differences in treponemal density and localization observed in our cohort.

IHC performance in our study was substantially superior to that reported by Müller et al. [5] (88% vs. 49%), even though both studies used a polyclonal antibody. Nevertheless, both studies pointed in the same direction in regard to a decline in treponemal density in advanced cases. In particular, biopsies from secondary syphilis frequently showed low treponemal density on IHC (15 of 36), rendering them prone to being overlooked.

Notably, in secondary syphilis, treponemas were entirely absent from the epidermis in almost 20% of the cases. This finding may explain the low IHC sensitivity reported in other studies [5].

These observations contrast those of Rosa et al. [16], who found treponemas in the epidermis in 80% of biopsies from secondary and tertiary syphilis. Although we cannot explain this discrepancy, previous authors emphasized potential pitfalls in interpretation of *Treponema pallidum* IHC, including staining of melanocytic dendrites [17] or of non-pathogenic spirochetes, e.g., in perianal lesions [18]. Moreover, not all of the studies discussed employed an identical IHC protocol [5,7,8].

Based on our observations, we conclude that an isolated evaluation of the epidermis in *Treponema pallidum* IHC is inadequate. Instead, systematic examination, including the endothelium of superficial and deep vascular plexuses, as well as perivascular and interstitial regions, is diagnostically crucial. The relatively frequent low treponemal density in secondary syphilis should alert pathologists that negative IHC results warrant cautious interpretation. In such cases, further diagnostic procedures—including clinical correlation, serology, and additional PCR assays—should be considered if syphilis remains suspected based on histology and immunophenotype.

Plasma cells are generally linked to be a histopathologic indicator for syphilis [19]; however, our study revealed that plasma cells were absent in 10% of biopsies and sparse in more than one-third of cases, particularly those from secondary syphilis. Immunostaining with CD138 was particularly helpful, increasing plasma cell detection by more than 30% and thereby enhancing histopathological suspicion of syphilis. Moreover, we demonstrated that granuloma formation can occur even in early disease stages, while an increase in the number of B-cells was a characteristic feature of secondary syphilis. Psoriasiform epidermal hyperplasia is considered typical of Syphilitic exanthema; however, in our cohort, psoriasiform features were not consistently present, with epidermal atrophy observed in one-third of secondary syphilis specimens.

Syphilis is referred to as “the great imitator” in the literature due to its diverse clinical manifestations [20]. Our observations confirm this impression, since syphilis diagnosis was considered only in 18 out of 38 cases (Figure 5). Illustrative examples of diagnostically challenging cases included a patient with an infiltrated reddish plaque in a newly tattooed area on the lower leg, followed by exanthematic spread; a patient with verrucous oral ulceration initially suspected to be carcinoma; and another with an abscess-like lesion on the abdomen. All these lesions were later confirmed to be primary syphilis.

Secondary syphilis can also pose significant diagnostic challenges. Although palmoplantar involvement of the exanthem is characteristic of secondary syphilis, it cannot be relied upon for diagnosis. Muna et al. demonstrated palmoplantar involvement in only 50% of patients with serologically confirmed syphilis [21]^,^ and in the present study, it was observed in a mere 15% of patients with secondary syphilis. Consequently, the exanthem was often difficult to distinguish from maculopapular exanthems of other etiologies, including HIV, EBV, or drug rash [22].

Not uncommonly, patients with syphilis are co-infected with HIV, which complicates the clinical and immunologic manifestations of the disease [23]. A study by Costa-Silva et al. [24] shows that *Treponema pallidum* PCR works equally for patients with and without HIV coinfection. In the present study, three patients had HIV coinfection, all secondary syphilis.

Biopsies from two showed low treponemal density, and in one more granulomatous case, no organisms were identified; however, all three cases yielded positive PCR results.

A similar case of nodular granulomatous secondary syphilis, confirmed by PCR on FFPE material, was reported by Falcinelli et al. [25]. Notably, in two of our patients, of whom we know that they were HIV-positive, we found only sparse plasma cells, even with CD138 staining, indicating that HIV coinfection may lead to atypical clinical and histological presentations [26,27].

In the case of unconventional clinical manifestation, clinicians are more likely to perform a skin biopsy, underscoring the importance of integrating histological characteristics, *Treponema pallidum* IHC and PCR on FFPE material in diagnostic practice. It must be noted that serological testing also has limitations or may not be performed if syphilis is not clinically suspected [28]. Additionally, our cohort demonstrated that patients benefited from multiple biopsies: for two patients, one biopsy was negative for both PCR and IHC, but a second biopsy from the same eruption taken concurrently was positive and confirmed secondary syphilis.

Limitations of our study include the retrospective design and the limited number of cases, some with small biopsy specimens that were exhausted after PCR procedures. Consequently, immunophenotyping of the inflammatory infiltrates could be performed in only 26 biopsies.

Moreover, our PCR results, obtained using a two-step approach, may not be directly comparable to those from other laboratories using different assays.

Although we employed a combination of two PCR assays, limited material precluded their application to all biopsies, preventing direct assay comparison in this study; this is planned for a subsequent project with new specimens. Finally, as with all morphological scoring systems, our assessments of treponemal density, infiltrate density, and cell populations remain somewhat subjective.

It is noteworthy that some of our FFPE biopsies were of considerable age, with some specimens up to 15 years old. As samples age, DNA quality declines, potentially impairing PCR performance and increasing the risk of false-negative results. However, in our cohort, no consistent association with age of the specimen could be identified. Other factors such as long fixation time in formalin probably also contribute to PCR failure and cannot be completely ruled out.

We acknowledge that application of statistical tests in this study bears the risk of multiple comparison bias, so significances should not be overestimated. Rather, they should be interpreted as tendencies needing further confirmation in subsequent prospective studies with larger cohorts.

In conclusion, *Treponema pallidum* IHC demonstrated in our cohort a slight advantage over PCR in the diagnosis of syphilis in FFPE skin biopsies, even with combining two PCR assays with different target genes. IHC is therefore extremely useful in routine diagnosis, particularly when accounting for variable treponemal density and distribution. Low Treponema density in about 40% and epidermal absence of organisms in nearly 20% of secondary syphilis biopsies highlight a substantial risk of overlooking treponemas on IHC; systematic assessment—including endothelium and perivascular areas—is therefore essential. A negative *Treponema pallidum* PCR does not exclude syphilis in FFPE skin biopsies, but PCR remains valuable in cases with atypical clinical features and equivocal IHC and/or serology—particularly in patients with repeated infections or altered immune status.

## Figures and Tables

**Table 1:** Demographical data, clinical features, clinical diagnosis and differential diagnosis, serological confirmation, molecular and immunohistochemical results of included patients/ and biopsies. NA: Not available; yr: Years; POS: positive; NEG: negative; f: female; m: male; IHC: Immunohistochemistry; PCR 1 [10,11], PCR 2 [12]; shadow: PCR 2 not performed.

Patient	Age (yr)	Sex	Site	Lesions	Clinical Diagnosis	Clinical Differential Diagnosis	Treponema Pall. IHC	PCR 1	PCR 2	Serological Confirmation	Lues Stage
1	58	m	Penis shaft	Skin-colored, asymptomatic nodus with erosion	Cyst	Exclusion of neoplasia	POS	POS		YES	1
2	41	m	Back, paravertebral	Trunk-centered exanthem	Scabies	Sweet syndrome	POS	POS		YES	2
3	28	m	Penis shaft	Fibrin covered ulceration	Lues	NA	POS	POS		YES	1
4	35	m	Penis	4 mm erosive plaque	Lues	Lymphogranuloma venerum	POS	POS		YES	1
5	52	m	Penis	7 mm red papule	Verruca vulgaris	Lues	POS	POS		YES	1
6	74	m	Penis, trunk	Trunk-centered exanthem, genital erosions	Unclear Exanthem	Lues, genital Herpes	POS	POS		YES	2
6	74	m	Back, paravertebral	Trunk-centered exanthem, genital fissure	Lues Reinfection	Drug rash	POS	POS		YES	2
7	40	m	Abdomen, trunk	Exanthem with papules	Pityriasis lichenoides	Lymphomatoid Papulosis	POS	POS		YES	2
8	44	m	Anal	NA	Ulcus molle	NA	POS	POS		YES	1
9	59	m	Right shoulder	NA	Lues	Scabies	POS	POS		YES	2
10	56	m	Back, paravertebral	Exanthem on the trunk and limbs, erosion on the palate	Lues	Psoriasis	POS	POS		YES	2
10	56	m	Back paravertebral	Exanthem on the trunk and limbs, erosion on the palate	Lues	Psoriasis	POS	NEG		YES	2
11	66	m	Tongue	Verrucous ulceration	Tongue carcinoma	Leukoplakia	POS	POS		YES	1
11	66	m	Tongue	Verrucous ulceration	Tongue carcinoma	Leukoplakia	POS	POS		YES	1
12	22	m	Inguinal	Confluent and erosive papules inguinal and perianal	Lues	NA	POS	POS		YES	2
13	48	m	Left shoulder	Infiltrated plaques, progressive	Sarcoidosis	NA	POS	POS (repetition cycle)		YES	2
13	48	m	Left shoulder	Infiltrated plaques, progressive	Sarcoidosis	NA	POS	POS (repetition cycle)		YES	2
14	51	m	Perianal	NA	Condyloma accuminatum	Lues	POS	POS		YES	1
15	52	m	NA	Trunk-centered exanthem	Allergic rash	Lues	POS	POS		YES	2
16	59	m	Oral	Oral ulceration	EBV infection	NA	POS	POS		YES	1
17	59	m	Trunk	Trunk-centered exanthem	Pityriasis rosea	Parapsoriasis	NEG	NEG		YES	2
17	59	m	Trunk	Trunk-centered exanthem	Pityriasis rosea	Parapsoriasis	NEG	POS		YES	2
18	52	m	Trunk, head	Multiple, asymptomatic nodules	NA	Leishmaniasis, Mycobacteriosis	POS	POS		YES	2
19	50	m	Anal	NA	Lues	Herpes genitalis	POS	POS		YES	1
20	47	m	Trunk, limbs	NA	Drug rash	Lues	POS	POS		YES	2
21	73	m	Trunk	Maculopapular exanthem	NA	NA	POS	POS		YES	2
22	28	m	Trunk	Erythematous macules	Lues	Drug rash	POS	POS		NA	2
23	55	m	Legs, abdomen	Isolated plaques	Sarkoidosis	Lues	POS	POS		YES	2
23	55	m	Legs, abdomen	Isolated plaques	Sarkoidosis	Lues	POS	POS		YES	2
24	52	m	Trunk	Trunk-centered, macular exanthem	Viral rash	NA	POS	NEG	NEG	YES	2
24	52	m	Trunk	Trunk-centered macular exanthem	Viral rash	NA	POS	NEG	NEG	YES	2
25	56	f	Trunk, limbs, palmoplantar	Macular exanthem on the trunk and limbs	Pityriasis rosea	Lues	POS	NEG	POS	YES	2
25	56	f	Trunk, limbs, palmoplantar	Macular exanthem on the trunk and limbs	Pityriasis rosea	Lues	POS	NEG	NEG	NA	2
26	47	m	Chin	Nodule on chin, onset of generalized exanthema	Lues	Leishmanionse	POS	POS		YES	1
27	40	m	Penis	Annular plaque	Erythema multiforme	Lues	POS	NEG	POS	YES	1
28	69	m	Chest	Papules on the trunk and palmoplantar	Psoriasis	Lues	POS	NEG	POS	YES	2
29	45	f	NA	NA	Urticarial rash	NA	POS	NEG	POS	YES	2
30	28	m	Gluteal	Papules perianal	Condylomata lata	Lues	POS	POS		YES	2
31	51	m	Abdomen	Generalized plaques, also perianal	Middermal elastolysis	Psoriasis	NEG	NEG	NEG	YES	2
31	51	m	Abdomen	Ulceration on scrotum, penis, oral cavity	Middermal elastolysis	Psoriasis	POS	NEG	POS	YES	2
32	46	m	Scrotum	NA	Unclear exanthem	NA	POS	POS		YES	2
33	21	m	Left axilla	Macular exanthem on the trunk and face	Viral rash	NA	POS	NEG	POS	YES	2
34	50	m	Left arm	Trunk-centered macular exanthem	Lues	Porokeratosis	POS	POS		YES	2
35	64	f	Left foot	Trunk-centered macular exanthem, including palmoplantar region	Grover’s disease	NA	POS	NEG	POS	YES	2
36	36	m	Back	Trunk-centered macular exanthem, also palmoplantar	Lues	Pityriasis rosea	POS	POS		YES	2
36	36	m	Left arm	Trunk-centered macular exanthem, also palmoplantar	Lues	Pityriasis rosea	POS	POS		YES	2
37	35	m	Back	Plaques on trunk and limbs	NA	Pityriasis rosea	POS	POS		YES	2
38	42	m	Right shoulder	Generalized macular exanthem	Drug rash	NA	POS	POS		YES	2

**Table 2:** Immunohistochemical Density and Localization of *Treponema pallidum* in Skin Biopsies of primary and secondary Syphilis: 1 (few), 2 (moderate), 3 (numerous). * The total number of specimens was 48, but one biopsy of primary syphilis was completely ulcerated, showing only dermal tissue; hence, 47 in the detailed assessment.

	Syphilis Stages			Statistics
	1 + 2	1	2	
**Epidermis** Total	47/48 * (97,9%)	11/12 * (91,7%)	**36/36 (100,0%)**	
Score 0	8 (17,0%)	1 (9,1%)	7 (19,4%)	
Score 1	17 (36,2%)	2 (18,2%)	15 (41,7%)	
Score 2	12 (25,5%)	2 (18,2%)	10 (27,8%)	
Score 3	10 (21,3%)	6 (54,5%)	4 (11,1%)	0,01
**Epidermis** Lower third	40/47 * (85,1%)	11/11 * (100,0%)	**29/36 (80,6%)**	
Score 1	17 (36,2%)	3 (27,3%)	14 (38,9%)	
Score 2	14 (29,8%)	3 (27,3%)	11 (30,6%)	
Score 3	9 (19,1%)	5 (45,5%)	4 (11,1%)	
**Epidermis** Middle third	**24/47 (51,1%)**	**7/11 (63,6%)**	**17/36 (47,2%)**	
Score 1	11 (23,4%)	1 (09,1%)	10 (27,8%)	
Score 2	11 (23,4%)	6 (54,5%)	5 (13,9%)	
Score 3	2 (04,3%)	0 (00,0%)	2 (05,6%)	
**Epidermis** Upper third	**9/47 (19,1%)**	**6/11 (54,5%)**	**3/36 (08,3%)**	**0,00260**
Score 1	3 (06,4%)	2 (18,2%)	1 (02,8%)	
Score 2	4 (08,5%)	3 (27,3%)	1 (02,8%)	
Score 3	2 (04,3%)	1 (09,1%)	1 (02,8%)	0,02382
**Dermis** Endothelial	**39/47 (83,0%)**	**10/11 (90,9%)**	**29/36 (80,6%)**	
Score 1	14 (29,8%)	0 (00,0%)	14 (38,9%)	
Score 2	12 (25,5%)	5 (45,5%)	7 (19,4%)	
Score 3	13 (27,7%)	5 (45,5%)	8 (22,2%)	0,0278
**Dermis** Perivascular	**38/47 (80,9%)**	**10/11 (90,9%)**	**28/36 (77,8%)**	
Score 1	19 (40,4%)	2 (18,2%)	17 (47,2%)	
Score 2	11 (23,4%)	3 (27,3%)	8 (22,2%)	
Score 3	8 (17,0%)	5 (45,5%)	3 (08,3%)	0,0139
**Dermis** Interstitial	**26/47 (55,3%)**	**9/11 (81,8%)**	**17/36 (47,2%)**	
Score 1	16 (34,0%)	3 (27,3%)	13 (36,1%)	
Score 2	7 (14,9%)	3 (27,3%)	4 (11,1%)	
Score 3	3 (06,4%)	3 (27,3%)	0 (00,0%)	0,00714

## Data Availability

Detailed study data is available from the corresponding author, Charlotte C. Fuchs, upon reasonable request.
